# Tremor Recurrence in MR-Guided Focused Ultrasound Thalamotomy for Essential Tremor: DBS vs. Re-lesion

**DOI:** 10.5334/tohm.1194

**Published:** 2026-05-27

**Authors:** Nur Walker-Pizarro, Jason L. Chan, Jun Yu, Justin D. Hilliard, Leonardo Almeida, Michael S. Okun, Matthew A. Remz

**Affiliations:** 1 Department of Neurology, Norman Fixel Institute for Neurological Diseases, University of Florida, Gainesville, Florida, United States; 2 Division of Neurology, Department of Medicine, University of British Columbia, Vancouver, British Columbia, Canada; 3 Department of Neurosurgery, University of Florida, Gainesville, Florida, United States

**Keywords:** Essential tremor, Deep Brain Stimulation, Ventral intermedius nucleus, MRI-guided focused ultrasound, Thalamotomy, Tremor recurrence

## Abstract

**Clinical vignette:**

A 59-year-old man with essential tremor (ET) and bilateral symptoms initially underwent left thalamic MRI-guided focused ultrasound (MRgFUS) at an outside institution to address tremor in the right upper extremity. Despite an initial improvement, the benefit waned within one year, and there was significant progression of right upper extremity tremor and disabling left upper extremity tremor.

**Clinical dilemma:**

Tremor recurrence following MRgFUS highlights a common clinical challenge in the management of medication-refractory ET. When tremor re-emerges, clinicians must determine whether to pursue re-lesioning or transition to an alternative surgical strategy such as deep brain stimulation (DBS).

**Clinical solution:**

Following a multidisciplinary evaluation, staged bilateral ventral intermediate nucleus of the thalamus DBS (VIM-DBS) surgery was performed and resulted in bilateral tremor control.

**Gap in knowledge:**

There is no consensus regarding the optimal management when tremor recurs post-MRgFUS. Guiding principles for balancing re-lesioning vs. DBS remain undefined in clinical practice.

**Highlights:**

Tremor recurrence following MR-guided focused ultrasound highlights the limitations of fixed lesioning in a progressive disorder. This case illustrates that staged bilateral deep brain stimulation can provide durable, adjustable tremor control even when stimulation is delivered within a previously focused ultrasound lesioned target.

## Clinical Vignette

A 59-year-old right-handed man with essential tremor (ET) was evaluated for surgical management. He had a long-standing history of action tremor predominantly affecting the hands. Tremor was right-predominant and progressively worsened over 10 years, later involving the lower extremities and head.

Treatment with clonazepam and propranolol provided mild benefit, and functional impairment persisted. He did not tolerate primidone or topiramate. Given predominantly unilateral functional disability and the patient’s preference for an incisionless approach, he underwent left MRI-guided focused ultrasound (MRgFUS) thalamotomy at an outside institution. The procedure was well-tolerated, with near-complete resolution of tremor on the treated side. By one-year post-MRgFUS, right-sided tremor worsened and disabling contralateral tremor emerged.

## Clinical Dilemma

Since FDA approval in 2016, MRgFUS thalamotomy has become an increasingly popular incisionless option for medication-refractory ET. Studies demonstrate robust short-term tremor reduction in appropriately selected patients, with outcomes in some comparative cohorts broadly similar to deep brain stimulation (DBS) [1,2,3,4]. Long-term durability, particularly in patients with bilateral or advancing symptoms, remains uncertain. With increasing MRgFUS adoption, clinicians more frequently encounter tremor recurrence, yet evidence beyond five years remains limited [5,6].

Several factors may contribute to symptom recurrence following MRgFUS. Loss of benefit does not necessarily imply technical failure but may instead reflect disease progression. Lesion characteristics, including location, size, and perilesional edema are known determinants of treatment response and durability [1,2,7,8]. Precise targeting of the ventral intermediate nucleus of the thalamus (VIM) is critical, and variability in targeting strategies may also impact outcomes [9].

Collectively, these uncertainties complicate decision-making when tremor re-emerges following an initially successful procedure. Recurrence should prompt reassessment of tremor phenomenology, lesion adequacy, and surgical sequencing.

## Clinical Solution

When tremor progresses following MRgFUS, management options include (1) medical therapy, (2) re-lesion, or (3) DBS. In patients with bilateral symptoms, DBS may be preferable due to the cumulative risks associated with lesion enlargement or creation of a second lesion [10].

**Figure 1:**
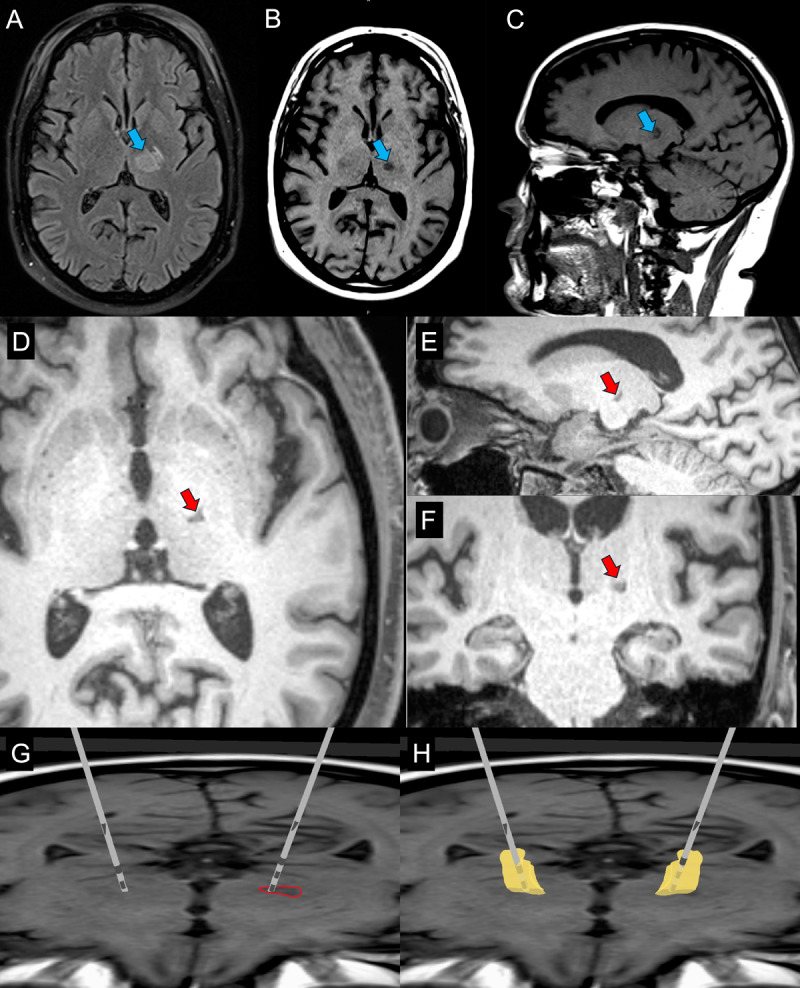
**MRgFUS VIM Thalamotomy and VIM-DBS Localization**. The MRgFUS lesion is visualized in the VIM thalamus on MRI on postoperative day 1 (blue arrows) demonstrating an adequate lesion on axial T2-FLAIR **(A)**, axial T1-weighted **(B)** and sagittal T1-weighted **(C)** images. The lesion is redemonstrated at 24 months post-MRgFUS (red arrows) on T1-weighted MRI images in the axial **(D)**, sagittal **(E)**, and coronal **(F)** planes. Following DBS surgery, post-op CT was fused to pre-operative MRI, and the DBS lead was reconstructed using Lead-DBS [38]. The DBS lead is demonstrated within the previously lesioned tissue (red outline) on 24-month post-lesion axial T1-weighted MRI **(H)**. The predicted VIM location (yellow), derived from the DISTAL atlas [39] and projected in MNI space, is superimposed on the same T1-weighted image **(G)**.

The MRgFUS lesion was reviewed on postoperative (Figure 1A–F) and pre-operative DBS planning MRIs (Figure 1G). Given the reasonable lesion size and location, and the presence of tremor without ataxia, the clinical team was worried that increasing lesion size may increase side-effect risk. Lesion enlargement carries a dose-dependent risk of imbalance, particularly in progressive ET and in patients who may later require bilateral surgical therapy [11]. Following multidisciplinary evaluation, he was deemed a suitable candidate for staged bilateral VIM-DBS, beginning with right VIM implantation to address the more severe left-sided tremor. DBS was selected to provide a durable, adjustable therapy given disease progression and bilateral lesional risks [10]. Although historical lesion risks include factors less applicable to MRgFUS (e.g., probe passage through normal cortex, modifying hemorrhage and infection risk), concerns remained regarding balance impairment with a larger and/or second lesion. The possibility of increasing the lesion size on one side and adding DBS on the other was considered; however, further lesioning was avoided due to concerns regarding adverse-events [11].

The patient opted for bilateral DBS, citing this as the safest option to maximize benefit and minimize long-term side effects. The ability to adjust DBS over time was appealing, given prior recurrence following a fixed lesion and limited options for further improvement.

Postoperatively, optimized right-sided DBS resulted in approximately 85–90% tremor improvement. Subsequently, the patient underwent left VIM-DBS for contralateral tremor. The second lead was placed on the previously MRgFUS-treated side (left VIM-DBS) at a 21-month interval following the first DBS procedure (Figure 1H). The Tremor Rating Scale (TRS) improved from a total motor score of 24 at baseline to 10 following bilateral VIM-DBS (27 months after right-sided implantation and 6 months after left-sided implantation).

Notably, therapeutic stimulation was achieved despite lead placement within a previously lesioned VIM. This suggests that the MRgFUS lesion may have preserved sufficient surrounding tissue to permit subsequent neuromodulation. Unlike prior reports emphasizing suboptimal lesion placement, this case demonstrates that DBS can be integrated into longitudinal care after MRgFUS, even when the initial lesion appears appropriately located and sized.

This case highlights the distinction between fixed lesional therapies and programmable neuromodulation in ET. While MRgFUS delivers a static intervention, DBS provides an adjustable platform responsive to disease progression. In progressive ET, this distinction becomes increasingly relevant as symptoms evolve beyond the durability of focal lesioning.

## Gap in Knowledge

Rather than representing a corrective response to a prior intervention, this case illustrates how surgical strategies for ET may need to adapt to a progressive disease trajectory.

Although VIM-DBS has been a longstanding therapeutic option for medication-refractory tremor, MRgFUS has emerged as an increasingly used alternative. ET is a progressive disorder, with longitudinal studies demonstrating annual worsening, approximately 1–5% depending on outcome measures [12,13]. While short-term MRgFUS outcomes are well described, long-term durability and recurrence management remain undefined [3,4,5,6,14,15,16,17,18,19,20,21,22].

Although short-term efficacy and safety appear comparable to unilateral DBS, tremor recurrence following MRgFUS occurs in approximately 10–15% of cases. Estimates vary by clinical phenomenology, follow-up duration, and cohort characteristics [18,23,24]. Given ET progression, tremor recurrence or emergence of ataxia is expected with longer follow-up; clinicians must disentangle the two before offering repeat intervention.

The contributions of lesion size and location to tremor recurrence remain a topic of research. While some authors associate larger lesions with more durable benefit, others have failed to demonstrate correlation between lesion metrics and long-term outcomes, suggesting that lesion characteristics alone may be insufficient to explain recurrence [16,20]. Variability in early post-procedural lesion measurements complicates outcome prediction, with some lesions reported as disappearing despite continued improvement [16,19,20,25]. Additionally, MRgFUS lesions may preserve more surrounding tissue than radiofrequency lesions, potentially allowing subsequent rescue DBS. This will require confirmation with post-mortem studies.

The recent FDA approval of bilateral MRgFUS introduces uncertainties regarding long-term durability and safety. Bilateral MRgFUS procedures may aim for smaller lesions, especially on one side, to improve safety. Whether this adjustment will be at the expense of optimal tremor control remains unknown [2,26].

Comparative studies suggest that bilateral VIM-DBS and unilateral VIM MRgFUS carry broadly comparable adverse effect burdens, albeit with different side-effect profiles. A higher prevalence of gait disturbances and paresthesias was observed in the MRgFUS cohort, whereas DBS patients more frequently reported speech disturbances [27,28,29]. Although historical concerns exist regarding cognitive outcomes following lesional therapies, early data from a small bilateral MRgFUS cohort suggest largely stable cognitive profiles [30].

The potential impact of prior lesion therapy on the outcome of subsequent DBS is not fully understood. Lesional therapies like MRgFUS can induce both structural and functional alterations within the treated brain area. There is evidence that lesions in the thalamus can, for example, lead to durable microstructural changes in connected pathways, including the cerebello-thalamo-cortical tract (CTCT) [31]. Prior studies of MRgFUS have reported regional changes in cerebral blood flow, gene expression, grey matter volume, and fractional anisotropy in related networks [32,33,34]. These alterations raise questions regarding how prior lesional therapy may influence DBS targeting accuracy, stimulation thresholds, and long-term responsiveness to therapy. Differences between lesion types (MRgFUS vs. radiofrequency) must also be considered.

This case illustrates the importance of timing when weighing different neurosurgical interventions. Decisions between MRgFUS and DBS, or between stages of a bilateral procedure, must consider evolving symptoms (tremor vs. ataxia), anatomical changes and potential cumulative risk. The literature provides limited guidance on the optimal interval between MRgFUS and subsequent DBS in ET, with reported rescue cases ranging from 4 to 24 months, and no established predictors of response [1,10,35,36].

Collectively, these uncertainties underscore the need for patient-tailored, longitudinal treatment strategies that account for disease progression, phenomenology, prior interventions, and the relative strengths of lesional versus neuromodulatory approaches. As a single-case experience, these observations cannot establish generalizability, and optimal sequencing and timing of MRgFUS and DBS remains unclear.

## Expert Commentary

MRgFUS has rapidly reshaped the surgical landscape of ET, particularly as its use expands to patients with bilateral symptoms, raising questions regarding long-term efficacy in a progressive disorder and how to proceed when tremor re-emerges despite an apparently adequate lesion size and location.

In patients with progressive bilateral symptoms, clinicians may consider re-lesioning the initial side and adding a contralateral lesion, but at what cumulative risk? If tremor continues to progress, might bilateral DBS or a lesion on one side and a DBS on the other offer greater long-term flexibility? As MRgFUS utilization grows, guidance is needed regarding sequencing, timing, and risk tradeoffs.

Tremor type may be an overlooked factor in treatment outcomes. Tremor disorders can present with overlapping phenomenology, with up to 50% of cases ultimately receiving a secondary diagnosis [13,37]. Accurate identification of tremor phenomenology is crucial to guide intervention. Diagnostic heterogeneity may therefore contribute to variable durability of lesional therapies and reinforces the importance of diagnostic reassessments when tremor recurs after neurosurgical intervention. This consideration becomes particularly relevant when interpreting apparent loss of efficacy following an otherwise technically successful intervention.

When tremor re-emerges following MRgFUS, DBS offers several advantages: reversibility, adjustable stimulation parameters, and a well-established safety profile for bilateral implementation [1,10].

This case demonstrates that prior lesioning does not necessarily preclude subsequent DBS benefit and may complement it. Historically, radiofrequency thalamotomy resulted in more extensive tissue disruption, and stimulation within or adjacent to lesioned tissue has been technically challenging. MRgFUS, in contrast, may be more amenable to subsequent DBS integration.

Interestingly, the active DBS contact used in this case was located outside the MRgFUS-lesioned area when visualized on the most recent follow-up MRI. We speculate that clinical benefit may have resulted because stimulation was applied outside the long-term lesioned zone. When considering DBS after lesion therapy, clinicians should consider whether to deliberately locate contacts intended for electrical stimulation outside of the lesioned region.

Several practical lessons emerge from this case. First, loss of benefit following MRgFUS should not be assumed to represent technical failure, particularly when imaging confirms adequate lesion size and location and tremor symptoms evolve. Second, prior lesional therapy does not necessarily preclude subsequent DBS targeting or clinical efficacy, and stimulation may be feasible within or adjacent to the previously lesioned area. Finally, in a progressive disorder such as ET, DBS may offer advantages when symptom evolution outpaces the durability of fixed interventions, though counterbalanced by the limitations of stimulation-induced side effects.

Prospective studies examining patient selection, sequencing, and timing of MRgFUS and DBS are needed to inform individualized longitudinal neurosurgical strategies in ET. From a clinical perspective, MRgFUS and DBS should not be viewed as mutually exclusive therapeutic modalities. Future work should focus not only on comparative efficacy, but also on defining patient-tailored strategies that preserve flexibility between lesioning and neuromodulation across the evolving disease course.
